# Reversible HIV-Associated Nephropathy and Concomitant Encephalomyelopathy as the Initial Presentation of Advanced, Untreated HIV

**DOI:** 10.7759/cureus.112634

**Published:** 2026-07-14

**Authors:** Mya Ya Minn, Muhammad Qasim Umer, Andrew Chisom Madu, Hannah Stacey, Anjali Sujith

**Affiliations:** 1 Nephrology, Mid and South Essex NHS Trust, Basildon, GBR; 2 Renal Medicine, Mid and South Essex NHS Trust, Basildon, GBR; 3 Renal Medicine, Basildon and Thurrock University Hospital, Basildon, GBR; 4 Radiology, Basildon and Thurrock University Hospital, Basildon, GBR

**Keywords:** end stage renal disease (esrd), hiv associated nephropathy (hivan), hiv encephalomyelopathy, paraplegia recovery, recovery of normal renal function

## Abstract

Human immunodeficiency virus (HIV) can cause severe multiorgan dysfunction when left untreated. Concurrent, severe renal and central nervous system manifestations as the primary presentation of advanced HIV are rare in modern clinical practice. We report a case of a young female presenting with dialysis-dependent acute kidney injury (AKI) from HIV-associated nephropathy (HIVAN) and paraplegia from HIV encephalomyelopathy, both of which demonstrated remarkable recovery following antiretroviral therapy (ART).

A 31-year-old female presented with a 1-week history of pyrexia, malaise, and rapidly progressive bilateral lower limb weakness. She is a PLHIV (people living with HIV) and was diagnosed seven years ago; she had defaulted from follow-up before starting ART. Laboratory investigations revealed stage 3 AKI, with serum creatinine rising from 403 umol/L on admission to 1112 umol/L within two weeks, alongside high-grade proteinuria. Serology confirmed a plasma HIV-1 RNA viral load of 3,440,753 copies/mL and a CD4 T-cell count below the limit of detection (<8 cells/uL). A renal biopsy demonstrated classic histopathological features of HIVAN, showing collapsing glomerulopathy. Magnetic resonance imaging (MRI) of the brain and spine demonstrated extensive, diffuse abnormal T2-weighted fluid-attenuated inversion recovery (T2/FLAIR) signal intensities involving the cerebral hemispheres, basal ganglia, brainstem, and a long segment of the cervical cord, consistent with an advanced neuro-axis injury. Cerebrospinal fluid (CSF) analysis and CSF culture ruled out opportunistic neuro-infections. The patient was initiated on regular haemodialysis and an intensive antiretroviral regimen consisting of dolutegravir/lamivudine, darunavir, and ritonavir, alongside co-trimoxazole prophylaxis until her CD4 count was over 200 cells/mm^3^. Over a five-month follow-up period, the patient demonstrated an impressive clinical response. Her plasma viral load decreased to 41 copies/mL, serial neuroimaging revealed substantial resolution of the extensive intracranial and spinal cord lesions, together with recovery of motor power to 5/5 in all 4 limbs, and recovery of renal function, which allowed for the successful cessation of long-term haemodialysis.

This case underscores that advanced HIVAN requiring renal replacement therapy and extensive HIV-related central nervous system pathology can be profoundly reversed with timely, potent antiretroviral therapy. Clinicians must maintain a high index of suspicion for HIV in patients presenting with unexplained concurrent multiorgan dysfunction, as early intervention can avert permanent end-stage organ failure and severe neurological disability.

## Introduction

Human immunodeficiency virus (HIV) infection can cause progressive end-organ damage, with HIV-associated nephropathy (HIVAN) and central nervous system (CNS) complications, such as HIV encephalopathy, representing two of its most severe manifestations [1]. HIVAN is a primary driver of renal impairment in individuals living with HIV, particularly impacting populations of African ancestry [2,3]. Historically, untreated HIVAN carried an exceptionally poor prognosis, rapidly advancing to end-stage renal disease (ESRD) within weeks or months [1].

Morphologically, HIVAN is characterised by a collapsing form of focal segmental glomerulosclerosis (FSGS), accompanied by microcystic tubular dilatation and profound interstitial inflammation [1,2,4]. This aggressive parenchymal collapse is triggered by a direct cytopathic mechanism resulting from HIV-1 replication within glomerular and tubular epithelial cells [1,5]. These findings establish the renal parenchyma as an independent, long-term cellular reservoir for the virus, which can form as early as primary infection [5].

Unchecked systemic viral replication also allows HIV to cross the blood-brain barrier, inciting localised neuroinflammation within microglia and macrophages. Clinically, this can progress to severe neurocognitive and motor deficits, including vacuolar myelopathy presenting as paraplegia. The widespread implementation of combination antiretroviral therapy (ART) has fundamentally transformed the management of these advanced complications [4]. Initiating ART slows the decline of the estimated glomerular filtration rate (eGFR), thereby reducing the incidence of chronic kidney disease (CKD) and preventing progression to permanent ESRD [6].

However, managing concurrent CNS disease introduces complex pharmacokinetic challenges because drug availability is often restricted by the blood-brain barrier [7]. To achieve viral suppression in the cerebral compartment, clinicians utilise the CNS Penetration-Effectiveness (CPE) ranking system to select antiretroviral drugs with a high capacity to penetrate the cerebrospinal fluid (CSF) [7].

While partial renal recovery and modest neurological stabilisation under targeted ART are well-documented [7,8], the simultaneous recovery of both dialysis-dependent renal failure and profound motor deficits remains exceedingly rare. This case report describes an extraordinary clinical course of a patient with advanced HIV who initially presented with severe renal failure requiring mechanical hemodialysis alongside complete paraplegia. Following the initiation of a highly effective antiretroviral regimen, the patient achieved renal recovery, permitting successful cessation of renal replacement therapy, coupled with full reversal of her paraplegia.

## Case presentation

History and presentation

A 31-year-old female presented to the emergency department with a 1-week history of pyrexia and generalised malaise. Over the days, she noted progressive bilateral lower limb weakness that significantly limited her mobility. She was noted to have spastic paraplegia with motor power of 1/5 and double incontinence. She had a known diagnosis of HIV infection of seven years duration, but had never initiated antiretroviral therapy (ART) and had been lost to local follow-up. Her baseline renal function was documented as entirely normal approximately 15 months prior to the current presentation.

Diagnostic evaluation

On admission (Day 1), initial laboratory evaluation revealed severe renal impairment. Over the subsequent two weeks, her renal function deteriorated rapidly. Urinalysis and quantification at presentation demonstrated significant proteinuria and albuminuria. Serum free light chains were elevated, but the kappa/lambda ratio was normal. The initial renal and biochemical markers are outlined in Table 1.

**Table 1:** Initial and peak renal biochemical parameters following admission

Parameters	Patient Values	Reference Range
Serum Creatinine (Day 1)	403 umol/L	45-95 umol/L
Peak Serum Creatinine (Week 2 )	1112 umol/L	45-95 umol/L
Urine Protein-to-Creatinine Ratio (UPCR)	884 mg/mmol	< 15 mg/mmol
Urine Albumin-to-Creatinine Ratio (UACR)	566 mg/mmol	<3 mg/mmol
Serum Kappa/Lambda Free Light Chain Ratio	0.86	0.26-1.65

An extensive autoimmune and vasculitic screen was conducted to rule out alternative causes of rapidly progressive glomerulonephritis following recommended diagnostic protocols. Antinuclear antibodies (ANA), extractable nuclear antigen (ENA) screen, anti-double-stranded DNA (anti-dsDNA), anti-cardiolipin antibodies, myeloperoxidase (MPO) antineutrophil cytoplasmic antibodies (ANCA), proteinase 3 (PR3) ANCA, anti-glomerular basement membrane (anti-GBM) antibodies, and anti-phospholipase A2 receptor (anti-PLA2R) antibodies were all within normal reference ranges or undetected.

Serological profiling confirmed advanced HIV-1 infection, with a combined p24 antigen/antibody test returning positive across primary and secondary testing kits. Immunophenotyping demonstrated profound immunosuppression and an extremely high viral load. These immunological and virological parameters are summarised in Table 2.

**Table 2:** Baseline immunophenotyping and virological markers

Parameters	Patient Values	Reference Range
Baseline Plasma HIV-1 RNA	3,440,753 HIV RNA copies/mL	Undetectable
Viral Load	6.54 viral load (log _10 _cps/ml)	-
Absolute CD 3 Count	178 cells/uL	No data
Absolute CD4 Cell Count	<8 cells/uL	No data
Absolute CD8 Count	149 cells/uL	No data
CD4/CD8 Ratio	< 0.10	No data

Screenings for Hepatitis B surface antigen, Hepatitis C antibodies, and syphilis serology were negative. Latent and opportunistic infection workups, including a 42-day mycobacterial blood culture, Toxoplasma antibodies, and *Borrelia burgdorferi* serology, were unremarkable.

Histopathological findings

To elucidate the aetiology of the AKI, a percutaneous renal biopsy was performed in week 2. The histopathological evaluation of the renal tissue is shown in Figures 1-2, which confirmed a diagnosis of HIV-associated nephropathy (HIVAN) characterised by no immune complex being identified ultrastructurally. Bowman's capsule dilatation and collapse of glomerular tufts showed collapsing glomerulopathy.

**Figure 1:**
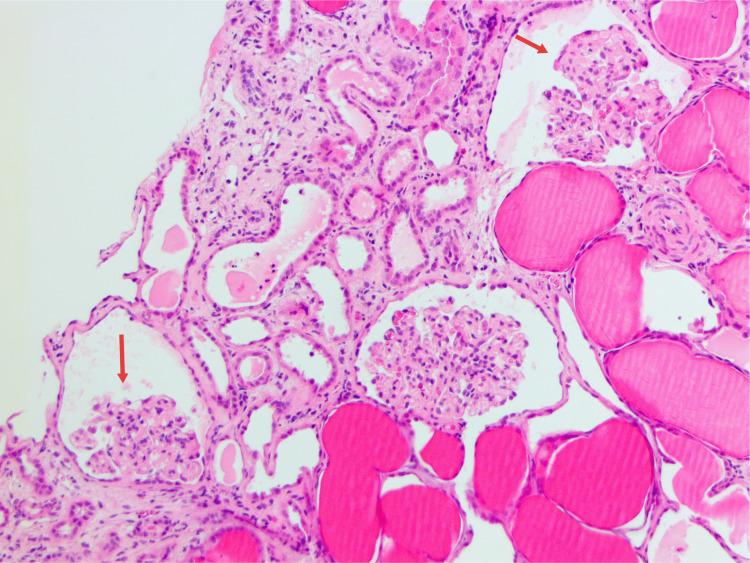
Collapsing glomerulopathy in HIV-associated nephropathy (H&E, x20) Both red arrows indicate collapsed glomerular tufts.

**Figure 2:**
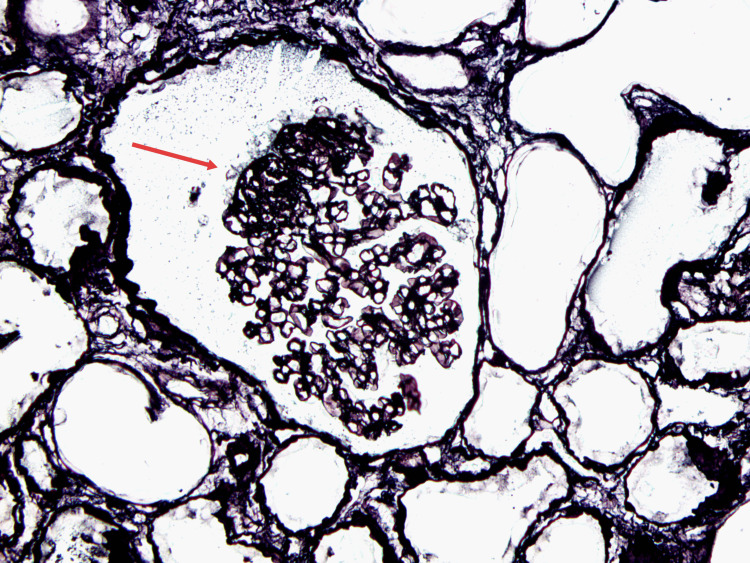
Jones methenamine silver stain x 40 The red arrow shows glomerular collapse with Bowman's space dilatation. Collapsing glomerulopathy is a typical presentation of HIV-associated nephropathy.

Neurological and radiological workup

Given the patient's progressive lower limb weakness, a comprehensive neurological workup was initiated in week 2. A lumbar puncture was performed to exclude opportunistic central nervous system infections. The cerebrospinal fluid (CSF) analysis demonstrated a clear appearance, a slightly elevated total protein of 0.56 g/L (reference range:0.15-0.45 g/L ), a normal lactate of 1.5 mmol/L (reference range: 1.1-2.4 mmol/L), and a normal glucose of 2.7 mmol/L (reference range: no data ). CSF Gram stain, bacterial cultures, and a 56-day mycobacterial culture yielded no growth. Extended CSF polymerase chain reaction (PCR) panels were completely negative for *Streptococcus pneumoniae*, *Neisseria meningitidis*, *Listeria monocytogenes*, JC virus, Cryptococcal antigen, Herpes Simplex Virus types 1 and 2, Varicella Zoster virus, and Enterovirus.

Neuroimaging revealed profound abnormalities across the central nervous system. An initial CT head scan in week 1 demonstrated confluent hypodensities throughout the periventricular and deep white matter bilaterally, which is shown in Figure 3.

**Figure 3:**
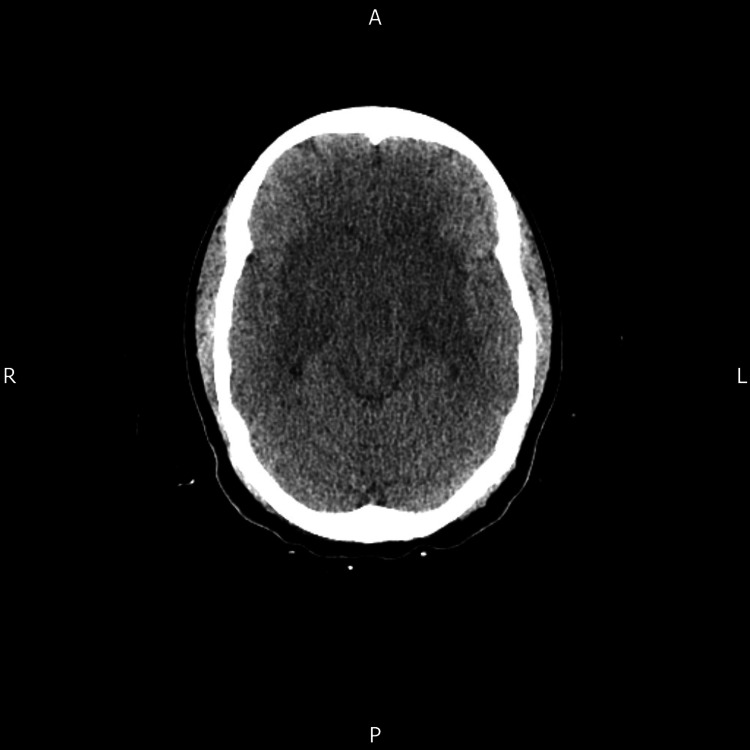
CT head showing confluent hypodensities throughout the periventricular and deep white matter bilaterally

A subsequent magnetic resonance imaging (MRI) of the brain was performed in week 1. The neuroimaging findings of the brain and spinal cord are displayed in Figure 4, which revealed extensive, diffuse abnormal high T2-weighted fluid-attenuated inversion recovery (T2/FLAIR) involving both temporal lobes, the thalami, basal ganglia, midbrain, and, to a lesser extent, the pons bilaterally.

**Figure 4:**
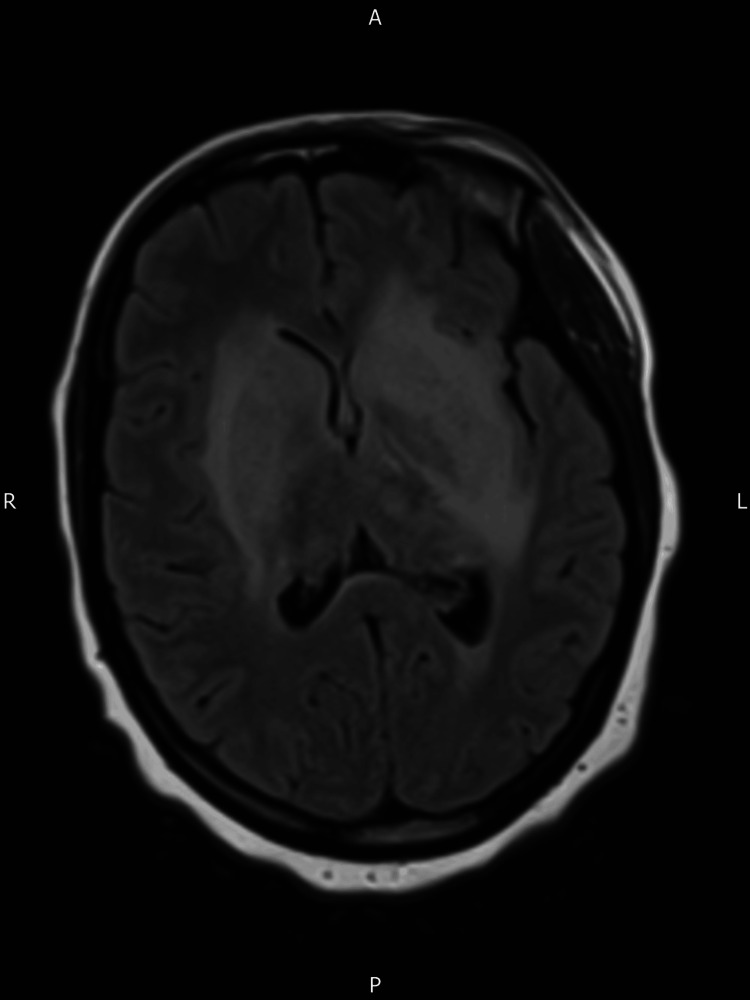
MRI FLAIR showing extensive and diffuse abnormal signal intensity involving both temporal lobes, the thalamus, and the basal ganglia FLAIR: fluid-attenuated inversion recovery

Figure 5 shows a long segment of abnormal cord signal and localised oedema involving the cervical spinal cord. These combined findings were highly characteristic of advanced HIV encephalopathy and HIV-related vacuolar myelopathy.

**Figure 5:**
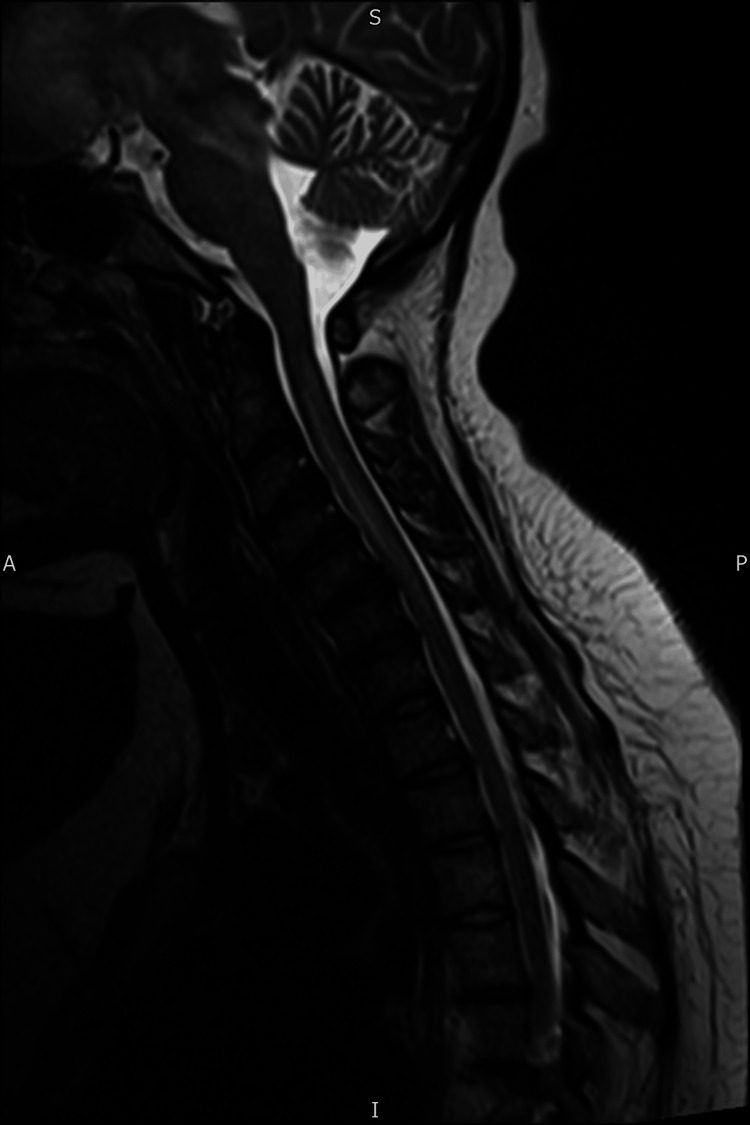
MRI spine sagittal T2 showing a long segment of abnormal cord signal involving the cervical cord

Management and clinical course

Due to worsening uremia and a peak serum creatinine of 1,112 umol/L, intermittent hemodialysis was initiated on Day 14 via a temporary dialysis catheter. The patient was commenced on a potent antiretroviral regimen consisting of Dovato (dolutegravir 50 mg/lamivudine 300 mg) once daily, supplemented with darunavir 800 mg and ritonavir 100 mg once daily to maximise viral suppression. The patient was monitored for immune reconstitution syndrome (IRIS).

The patient tolerated the intensive medical regimen well and was referred for ongoing neuro-renal rehabilitation.

Follow-up and outcome

The patient demonstrated an extraordinary recovery over the subsequent five months. Follow-up MRI imaging of the head and spine performed at Week 20 post-admission revealed substantial interval resolution of the extensive abnormal signal alterations across the cerebral hemispheres, thalami (Figure 6), basal ganglia, brainstem, and cervical cord (Figure 7).

**Figure 6:**
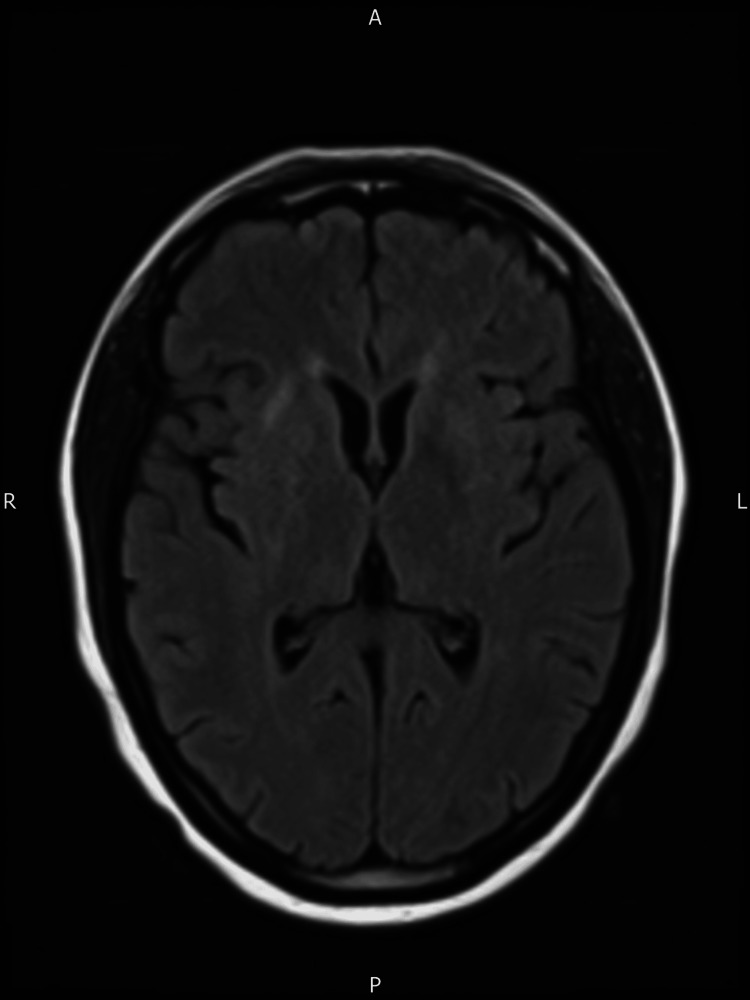
MRI head FLAIR showing interval improvement of diffuse cerebral signal abnormality involving the cerebral hemispheres, thalamus, and basal ganglia FLAIR: fluid-attenuated inversion recovery

**Figure 7:**
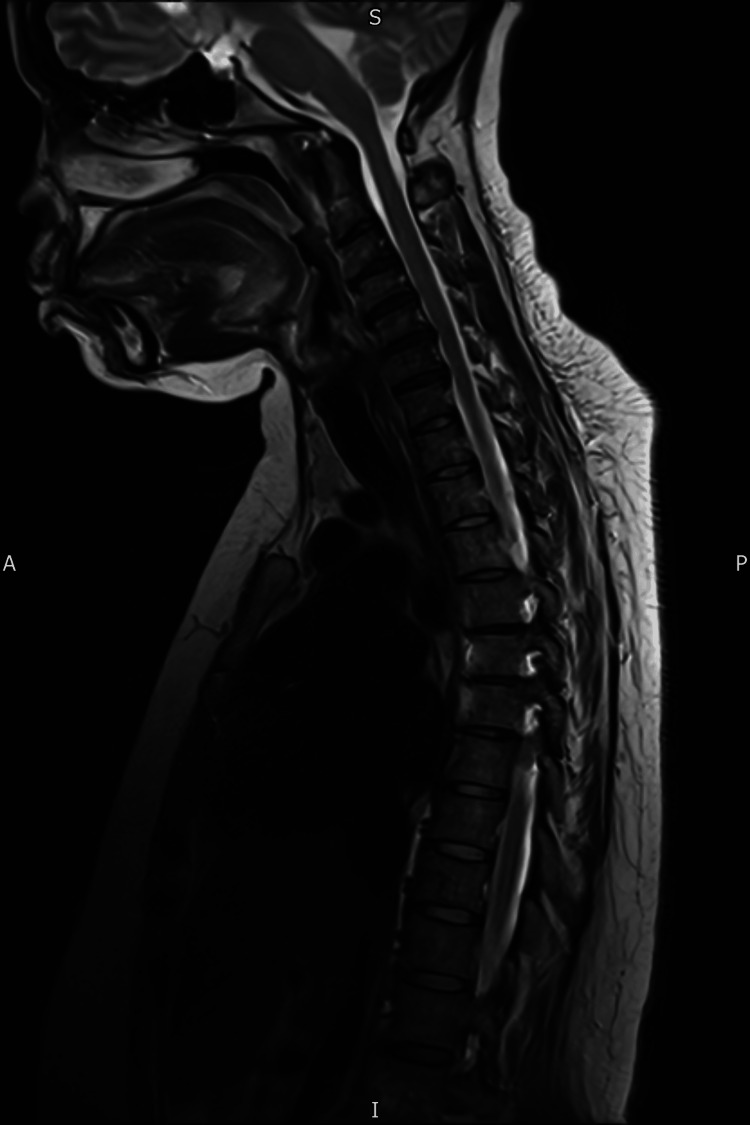
MRI spine T2 sagittal showing significant interval resolution of previously noted abnormal cervical cord signal and cord oedema

Concurrently, her renal parenchymal function and virological status recovered progressively. Her urine output increased steadily, and her serum creatinine values began to decrease out of proportion to dialysis clearance. The patient was successfully weaned off renal replacement therapy, receiving her final session of hemodialysis at Week 15 post-admission. Repeated virological and renal laboratory assessments performed at Week 20 demonstrated successful recovery parameters (Table 3).

**Table 3:** Follow-up renal and virological laboratory parameters at Week 20 post-admission

Parameters	Patient Values ( week 20 )	Reference Range
Serum Creatinine	251 umol /L	45-95 umol/L
Plasma HIV-1 RNA	40 HIV RNA copies/mL	Undetectable
Viral Load	1.61 viral load (log_10 _cps/ml)	-

Table 4 summarises the clinical course of the patient. 

**Table 4:** Summary of clinical course HIVAN: HIV-associated nephropathy; ART: antiretroviral therapy

Summary Timeline	Clinical Course
Week 1	Presented feeling unwell, with AKI 3 and paraplegia
Week 1	MRI showed advanced HIV encephalopathy and HIV-related vacuolar myelopathy
Week 2	Kidney biopsy showed HIVAN
Week 2	Haemodialysis was started
Week 4	ART was commenced
Week 20	Recovery of renal function
Week 20	Recovery of paraplegia and resolution of MRI abnormalities

## Discussion

The striking multisystem recovery observed in this patient underscores the profound capacity of combination ART to reverse the advanced, life-threatening complications of HIV. The patient’s initial presentation of severe, dialysis-dependent renal failure reflects the classic clinical trajectory of untreated HIVAN, an aggressive lesion characterised by the collapsing variant of focal segmental glomerulosclerosis (FSGS) and microcystic tubular dilatation [1,4]. Pathogenetically, this collapse is driven by direct HIV-1 infection and active viral transcription within glomerular podocytes and tubular epithelial cells, establishing the kidney as a distinct, long-term anatomical reservoir for the virus [5]. While HIVAN historically led to irreversible ESRD within weeks, the prompt initiation of ART halts active viral replication within this renal compartment, mitigating local inflammation and allowing cellular remodelling [2,3,5]. Large-cohort data confirm that ART significantly alters the natural history of HIV-related kidney disease by slowing the decline of the eGFR and reducing CKD progression [8].

Simultaneously, the complete resolution of the patient's paraplegia highlights the critical importance of selecting antiretroviral regimens with favourable pharmacokinetics capable of traversing the blood-brain barrier. Advanced CNS manifestations, such as HIV encephalopathy and vacuolar myelopathy, arise from localised neuroinflammation and viral replication within microglia and macrophages. Reversing severe neuro-axial motor deficits depends heavily on achieving robust viral suppression within the cerebral compartment. This clinical challenge is addressed by utilising the CPE ranking system, which grades individual antiretrovirals based on their chemical properties and CSF concentrations [7]. Regimens with high cumulative CPE scores are strongly correlated with lower CSF viral loads and improved neurocognitive outcomes [7].

ART were categorised into one of 3 categories: 0.0 (low: relatively poor estimated CNS penetration), 0.5 (medium: intermediate estimated CNS penetration), or 1.0 (high: relatively good estimated CNS penetration). The ranks are summarised in Table 5 [9].

**Table 5:** Antiretroviral central nervous system penetration scoring system CPE: central nervous system penetration-effectiveness score [7]

Drug class	CPE Score		
	1	0.5	0
Nucleoside reverse transcriptase inhibitor	Abacavir, Zidovudine	Emtricitabine, Lamivudine, Stavudine	Adefovir, Zalcitabine, Didanosine, Tenofovir
				Non-nucleoside reverse transcriptase inhibitor	Delavirdine, Nevirapine	Efavirenz	-
								Protease inhibitor	Amprenavir-r, Darunavir, Fosamprenavir-r, Lopinavir-r, Indinavir-r	Amprenavir, Atazanavir, Atazanavir-r, Fosamprenavir, Indinavir	Nelfinavir, Ritonavir, Saquinavir, Saquinavir-r, Tipranavir-r
				Integrase inhibitor	-	Elvitegravir, Raltegravir	-				
Entry inhibitors	Vicriviroc, Maraviroc	-	Enfuvirtide								

In this case, the dual recovery of haemodialysis dependence and profound paraplegia demonstrates that even when advanced end-organ collapse appears terminal, an optimised antiretroviral regimen can achieve dramatic, synergistic tissue recovery across both the renal and central nervous systems.

## Conclusions

This case highlights that clinicians must maintain a high index of clinical suspicion, performing routine HIV testing in any patient presenting with unexplained AKI or atypical neurological symptoms, regardless of a young age or an apparent low-risk profile. When advanced infection is suspected, performing an early renal biopsy is essential to definitively confirm the diagnosis, rule out concurrent immune-complex glomerulonephritides, and accurately quantify the degree of chronic interstitial fibrosis and tubular atrophy to guide the long-term prognosis. Crucially, severe presentations of HIV-associated nephropathy that require acute renal replacement therapy should not be viewed as absolute indications of irreversible end-stage renal disease, as robust organ recovery and recovery of renal function remain achievable. Ultimately, combination antiretroviral therapy remains a potent primary therapeutic modality capable of simultaneously halting viral replication, restoring native kidney function, and reversing profound cytopathic injury across multiple organ systems, including extensive intracranial and spinal cord lesions.
